# Polychromatic neutron phase-contrast imaging of weakly absorbing samples enabled by phase retrieval

**DOI:** 10.1107/S1600576723003011

**Published:** 2023-05-09

**Authors:** Maja Østergaard, Estrid Buhl Naver, Anders Kaestner, Peter K. Willendrup, Annemarie Brüel, Henning Osholm Sørensen, Jesper Skovhus Thomsen, Søren Schmidt, Henning Friis Poulsen, Luise Theil Kuhn, Henrik Birkedal

**Affiliations:** a Department of Chemistry and iNANO, Aarhus University, Gustav Wieds Vej 14, Aarhus, Denmark; b Department of Energy Conversion and Storage, Technical University of Denmark, Fysikvej 310, Kongens Lyngby, Denmark; c Laboratory for Neutron Scattering and Imaging, Paul Scherrer Institute, Villigen, Switzerland; d Department of Physics, Technical University of Denmark, Fysikvej 307, Kongens Lyngby, Denmark; e European Spallation Source ERIC, PO Box 176, Lund, Sweden; f Department of Biomedicine, Aarhus University, Wilhelm Meyers Allé 3, Aarhus, Denmark; g Xnovo Technology ApS, Galoche Alle 15, 1, Køge, Denmark; Australian Nuclear Science and Technology Organisation, Lucas Heights, Australia

**Keywords:** phase-contrast imaging, neutron imaging, bone, tomography, phase retrieval, polychromatic neutrons

## Abstract

The use of a phase-retrieval technique for propagation-based phase-contrast neutron imaging with a polychromatic beam is demonstrated. This enables imaging of samples with low absorption contrast and/or improving the signal-to-noise ratio to facilitate *e.g.* time-resolved measurements. A metal sample, designed to be close to a phase pure object, and a bone sample with canals partially filled with D_2_O were used for demonstrating the technique. These samples were imaged with a polychromatic neutron beam followed by phase retrieval. For both samples the signal-to-noise ratios were significantly improved and, in the case of the bone sample, the phase retrieval allowed for separation of bone and D_2_O, which is important for example for *in situ* flow experiments. The use of deuteration contrast avoids the use of chemical contrast enhancement and makes neutron imaging an interesting complementary method to X-ray imaging of bone.

## Abstract 2

Neutron imaging enhanced by the retrieval of propagation-based phase contrast is described.

## 1. Introduction

There is a continued need for improved 3D imaging methods to analyse complex multi-length-scale structures of a plethora of technologically or biologically important materials. In this connection, neutron imaging is of interest due to the good penetration capabilities of neutrons, the non-continuous dependence of interaction cross sections with atomic number and the possibilities for adjusting material contrast by controlling isotope compositions.

The primary principle of image formation with neutron imaging is attenuation contrast from absorption. This is not always the best way to provide contrast, especially for samples consisting of materials with very similar absorption cross sections or very low absorption. For these cases, it is useful to apply the principle of phase contrast that harnesses the real part of the refractive index. Phase-contrast imaging can be performed in several ways using various kinds of interferometry (Pushin *et al.*, 2017 ▸; Strobl *et al.*, 2019 ▸) or propagation-based phase contrast (Fiori *et al.*, 2006 ▸; Paganin *et al.*, 2023 ▸). The benefit of the latter is that no gratings or other specialized elements are needed to measure the phase shift. Therefore, propagation-based phase-contrast imaging has evolved into a very powerful tool in X-ray imaging (Alloo *et al.*, 2022 ▸; Bidola *et al.*, 2017 ▸; Wieland *et al.*, 2021 ▸; Yu *et al.*, 2021 ▸) but has only been investigated for neutrons in a very few cases (Allman *et al.*, 2000 ▸; Jacobson *et al.*, 2004 ▸; Lehmann *et al.*, 2005 ▸; McMahon *et al.*, 2003 ▸).

When neutrons pass through a sample, they are refracted due to local variations in the refractive index. Features in the sample with different refractive indices will act as neutron lenses that either focus or diverge the neutrons from that point in the sample. These changes in the neutron directions are small and thus difficult to observe at short distances between the sample and detector, but they do become progressively easier to detect when increasing the distance between the sample and detector. The build-up of phase contrast requires a coherent beam, meaning that the beam divergence must be smaller than the changes caused by refractive features. Propagation-based phase-contrast imaging only gives relative information about the sample, which means that a phase-retrieval technique is required in order to obtain the projected density of the sample (Paganin *et al.*, 2023 ▸).

Here we explore propagation-based phase-contrast imaging, first in a model sample with very low absorption and secondly in bone, which is a hierarchically structured material.

Bone is replete with blood vessels and cells situated in lacunae interconnected by canaliculi only a few hundred nanometres in diameter (Wittig *et al.*, 2022 ▸). Together, these form a vast fluid-containing network. The transport of fluid in bone is very important since the fluid contains nutrients, signal molecules and ions essential for the entire body (Cowin & Cardoso, 2015 ▸). In addition, liquid transport is proposed to be the main mechanism of stress sensing in bone, suggesting that the osteocytes sense changes in shear liquid flow through the canaliculi (van Tol *et al.*, 2020 ▸; Robling & Bonewald, 2020 ▸; Burger & Klein-Nulend, 1999 ▸). Our understanding of liquid transport through the complex porous network of large canals [mean Harversian canal diameter 85 µm (in iliac crest) (Busse *et al.*, 2013 ▸)] and smaller channels – all the way down to the cellular level – remains incomplete. The potential of neutron imaging to afford insights into liquid transport in bone is thus highly interesting, since neutron imaging enables contrast variation by deuteration. The field of neutron imaging of bone tissue is developing (Törnquist *et al.*, 2020 ▸; Guillaume *et al.*, 2021 ▸), but methods providing improved signal-to-noise ratios and/or faster measurements remain in high demand.

Neutron propagation-based phase-contrast imaging has been demonstrated for near-monochromatic neutron beams (Paganin *et al.*, 2023 ▸), but being able to harness the higher flux accessible with pink or even white neutron beams [see for example McMahon *et al.* (2003 ▸)] would increase the efficiency and applicability of the method significantly, which is indeed the aim of the current contribution.

## 2. Experimental

### 2.1. Metal sample

**Figure 1:**
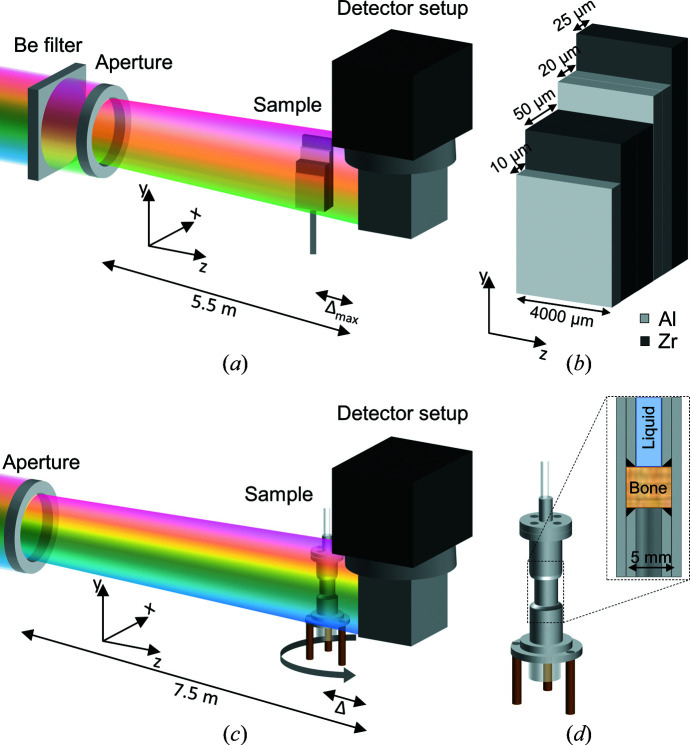
The experimental setup for neutron propagation-based phase-contrast measurements with either a pink or a white beam. (*a*) A schematic diagram of the experimental setup for a high phase contrast/low absorption contrast metal sample and a pink beam, showing the position and angle of the sample relative to the detector and aperture. The coordinate system refers to the phase-retrieval algorithm. (*b*) A diagram of the metal foil sample consisting of Al (light grey) and Zr (dark grey). Note the width and thicknesses are not on the same scale. (*c*) A diagram of the setup for white-beam imaging of bone, showing the position of the sample relative to the aperture and detector. (*d*) A schematic diagram of the sample holder, which could be connected to a pump at the top in order to force liquid through the bone sample that is placed in the middle. A reservoir is present in the bottom to collect liquid. The inset shows a vertical cut through the sample holder, with the sample in the middle packed between O rings and metal pipes holding it in place. The top pipe contains liquid while the bottom pipe is empty. The colour variation across the beams in panels (*a*) and (*c*) is to indicate relative wavelength ranges and not to suggest a variation in wavelength across the beam.

To validate the use of phase retrieval of propagation-based phase-contrast neutron imaging with a polychromatic neutron beam, a sample was designed to have a good neutron phase-contrast signal but weak neutron absorption contrast. The sample was made of Al and Zr sheets (coherent scattering cross section and thermal neutron absorption cross section of 6.44 and 0.185 barn, respectively, for Zr and of 1.495 and 0.231 barn, respectively, for Al; 1 barn = 10^−28^ m^2^) with thicknesses of 10 and 25 µm, respectively, such that various thicknesses were obtained [Figs. 1 ▸(*a*) and 1 ▸(*b*)]. The foils were cut into three pieces, each of width 4 mm and heights ranging between 8 and 11 mm, and assembled in a staircase configuration as shown in Fig. 1 ▸(*b*).

### 2.2. Phase retrieval

For neutron absorption imaging the projection images are formed according to the Beer–Lambert attenuation law. In phase-contrast imaging this is not sufficient to describe the full contrast mechanism. Instead, the projection 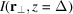 at a sample-to-detector distance *z* = Δ in the Fresnel regime and as a function of two-dimensional positional coordinates in the plane 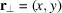 can be described as (Paganin *et al.*, 2023 ▸)

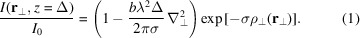

Here *I*_0_ is the beam intensity without sample, *b* is the bound coherent scattering length, λ is the wavelength of the neutron beam, 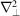 is the Laplacian in the *xy* plane, σ is the total neutron cross section and 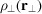 is the number density of atoms described by a vector in the *xy* plane.

Phase retrieval from measurements using a single sample-to-detector distance developed for use in X-ray propagation-based phase contrast by Paganin *et al.* (2002 ▸) is sometimes referred to as Paganin filtering. It has recently been adapted for use in neutron phase-contrast imaging (Paganin *et al.*, 2023 ▸).

The goal of phase retrieval is to obtain the number density 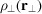 of atoms of a single-material sample given the propagation-based phase-contrast image 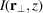 . Paganin *et al.* (2023 ▸) showed that this could be obtained by

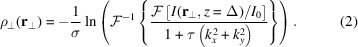

Here, 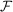 and 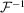 denote the spatial Fourier and inverse Fourier transforms, respectively, (*k*_*x*_, *k*_*y*_) are Fourier-space frequencies corresponding to (*x*, *y*), and τ is

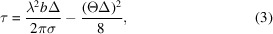

where Θ is the beam divergence. The core of this expression is the Fourier-space low-pass filter 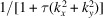 , which depends solely on the parameter τ that determines the strength of the phase-contrast signal. The result of equation (2) is the number density of atoms for a given volume in the sample under the assumption that the sample consists of a homogeneous material.

It is important that τ > 0, to avoid the denominator in equation (2) vanishing. This is achieved primarily through collimation of the neutron beam. From equation (3) we get the collimation condition

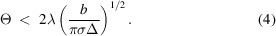

This presents a trade-off between having a divergent beam with higher neutron intensity, and thus better statistics, but worse phase contrast, and having a more coherent beam with reduced signal-to-noise ratio but a better phase-contrast signal. The divergence in the experiments reported herein is described below.

Equation (2) is only valid for a monochromatic beam. For a polychromatic, pink or white beam, the wavelength dependence should in principle be treated explicitly, which would require an energy-sensitive detector. Paganin *et al.* (2023 ▸) derived an approximate treatment employing effective spectrally averaged quantities as shown in equation (5),

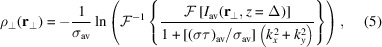

where the spectrally averaged quantities *I*_av_, σ_av_ and (στ)_av_ are defined as

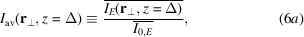




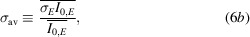




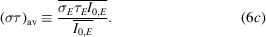

Here, *I*_0,*E*_ is the energy spectrum of the emitted neutrons. We used this formulation for all phase-retrieval calculations in the present work.

### 2.3. Neutron imaging of metal test sample with pink beam

Neutron imaging measurements on the metal test sample were performed on the Imaging with Cold Neutrons beamline (ICON) at the Paul Scherrer Institute (PSI), Switzerland (Kaestner *et al.*, 2011 ▸). The beam was modified with a Be filter to suppress neutrons with wavelengths below 4 Å, followed by a 10 mm circular aperture to increase spatial coherence further [Fig. 1 ▸(*a*)]. This led to a wavelength range of 4–9 Å, with a weighted mean wavelength of 5.35 Å. The detector was a CCD camera with a 20 µm thick Gadox scintillator and a pixel size of 13.5 × 13.5 µm^2^. The flux incident on the sample was calculated from the open-beam measurements to be 4.1 × 10^6^ n cm^−2^ s^−1^. Radiographs were recorded of the metal foil sample with an exposure time of 15 s. The distance between the aperture and the detector was 5.5 m, resulting in a beam divergence of Θ = 1.8 × 10^−3^ (*L*/*D* = 550, with *L* being the aperture-to-sample distance and *D* the aperture size), which fulfils equation (3) for all sample-to-detector distances. The sample was initially placed at a distance Δ = 19 mm from the detector. Radiographs were acquired at increasing sample-to-detector distances at every 5 mm up to an end position of Δ = 189 mm to map the transition from an absorption-dominated to a phase-contrast-dominated regime. A schematic diagram of the setup is shown in Fig. 1 ▸(*a*).

**Figure 2:**
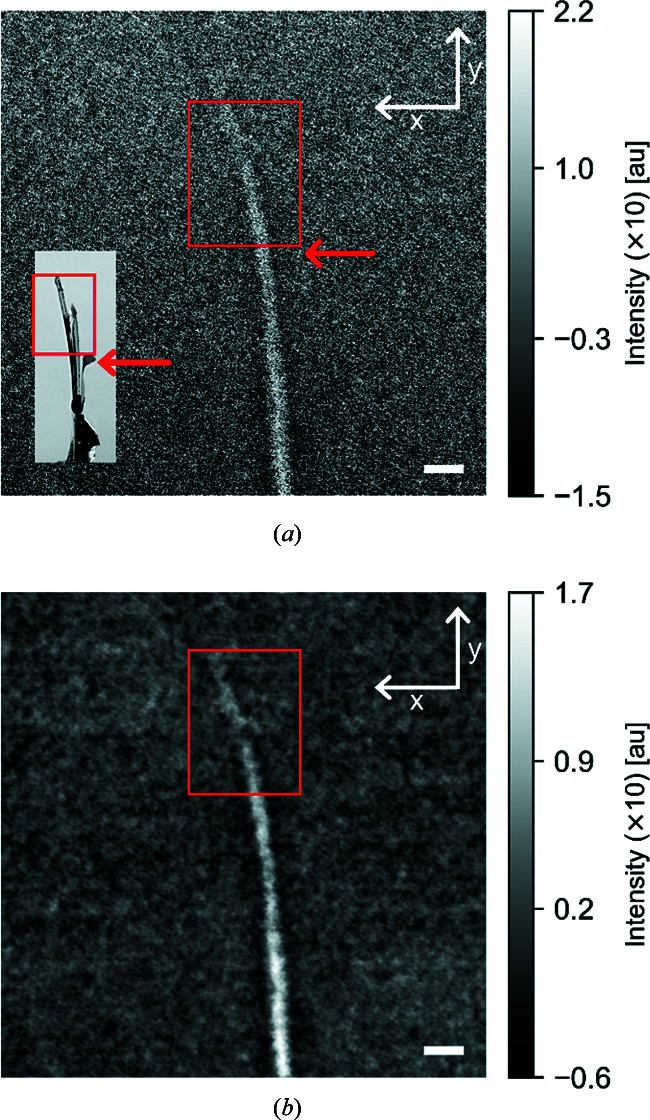
Neutron radiographs of the metal foil sample at a sample-to-detector distance Δ = 189 mm. The arrows point to the sample. Scale bars represent 1 mm. Radiographs (corrected as described in the *Experimental* section[Sec sec2]) are shown (*a*) without phase retrieval and (*b*) after phase retrieval. The inset in panel (*a*) shows a photograph of the metal foil sample. The red boxes indicate the same region in the photograph and radiographs. Note that the photograph is not recorded in the exact same orientation as the radiographs and the arrows do not point to exactly the same position in the sample and the signal.

The collected data were flat-field and dark-field corrected. Before further analysis, diagonal stripes introduced by the Be filter and a background gradient were removed from the data. The stripes were removed by masking out the regions of Fourier space corresponding to their frequency. The linear background gradient was removed by normalizing to the air signal outside the sample. A radiograph of the metal foil sample after correction is shown in Fig. 2 ▸(*a*).

Phase retrieval based on the assumption of a monochromatic beam with wavelength given by the mean of the spectrum resulted in the image shown in Fig. 2 ▸(*b*). Phase retrieval assumes that the sample consists of a homogenous material but this assumption has been found not to be essential. Beltran *et al.* (2010 ▸) demonstrated that the neutron cross section and scattering length can be chosen to correspond to the material of interest, which only locally blurs the boundaries between the material of interest and other materials (Beltran *et al.*, 2010 ▸, 2011 ▸). Thus, the neutron cross section and scattering length were calculated from a volume-weighted mean of the constants for Al and Zr, to give results of σ_eff_ = 5.04 × 10^−28^ m^2^ and *b*_eff_ = 6.10 × 10^−15^ m, respectively.

### 2.4. White-beam neutron imaging of bone samples

#### 2.4.1. Bone samples

Human bone was obtained from the Body Donation Programme at the Department of Bio­medicine and Health, Aarhus University. Required permission was obtained from the Scientific Ethical Board of the Region of Central Denmark (1-10-72-113-15).

Rods of human femoral bone (ø = 5 mm) from one individual were extracted using a hole saw so the sample long axis coincided with the load-bearing axis of the bone. Therefore, the vascular canals were mainly oriented parallel to the flow direction in the custom-built aluminium sample holder [Fig. 1 ▸(*b*)]. Two bone samples were extracted; one composed of both normal cortical bone and more porous cortical bone, giving rise to large void spaces, and one comprising normal cortical bone only, so the only void spaces visible at this resolution are blood vessels. In addition to the Harversian canals, cortical bone contains a cellular network with lacunae of 1–10 µm linear dimensions interconnected by canaliculi that are a few hundred nanometres in diameter, which cannot be observed at the resolution used (Wittig *et al.*, 2022 ▸).

#### 2.4.2. X-ray imaging of bone samples

Prior to the neutron measurements, the bone samples were investigated by X-ray microtomography using an Xradia 620 Versa X-ray microscope (ZEISS, Germany) with an accelerating voltage of 50 kV, a power of 4.5 W and an LE2 (low-energy) filter. A total of 3201 projections with an exposure time of 8 s each were collected while rotating the sample 360°, providing an isotropic voxel size of 5.5 × 5.5 × 5.5 µm^3^ obtained with an optical magnification of 0.4× and no pixel binning. The source-to-sample distance was 17 mm and the sample-to-detector distance was 88.8 mm. The reconstructions were conducted in the *Reconstructor Scout-and-Scan* software (Version 16.1.13038, ZEISS, Germany) using a cone-beam-adapted filtered back-projection (FBP) algorithm, rotated and tilted to match the neutron data using *ImageJ* (Schneider *et al.*, 2012 ▸), and exported to *Dragonfly* [Version 2021.3, Object Research Systems (ORS) Inc., Montreal, Canada] for segmentation and rendering.

#### 2.4.3. Neutron imaging of bone samples

Propagation-based phase-contrast neutron imaging of the bone samples was also performed on the ICON beamline at SINQ, PSI. Using a white beam and a 10 mm aperture [Fig. 1 ▸(*c*)], both 2D radiography and 3D tomography data were collected with 80 s exposure times and an isotropic voxel size of 13.5 × 13.5 × 13.5 µm^3^. The distance between the aperture and the detector was 7.5 m [Fig. 1 ▸(*c*)], leading to a beam divergence of Θ = 1.3 × 10^−3^ (*L*/*D* = 750), which fulfils equation (4). The first tomography data set was collected for the bone sample not containing bigger void spaces without the custom-built aluminium sample holder, thus allowing for a small sample-to-detector distance of Δ = 7 mm. A second tomography data set was collected for the more porous bone sample mounted in a custom-built aluminium sample holder [Fig. 1 ▸(*d*)]. Consequently, the sample-to-detector distance was larger than before, Δ = 48 mm. D_2_O (Eurisotop) was pumped using the lowest-possible pump setting (∼1 ml h^−1^) while the liquid transport was followed by time-resolved radiography. Afterwards, with the pump off but still connected, tomography data were collected. Both tomography data sets were obtained with 625 regularly spaced projections covering 0–360° and an exposure time of 80 s per projection.

The projections were normalized and Paganin single-distance phase retrieval was performed under the assumption of a monochromatic beam with a mean wavelength of 3.1 Å (see below for a discussion of the choice of wavelength in phase retrieval). For this purpose, the neutron cross section and scattering length were each estimated as the mean of the constants for the constituents of hy­droxy­apatite. For ring removal, both horizontal and vertical stripes were removed from the sinograms (Münch *et al.*, 2009 ▸) and the resulting projections were reconstructed in MATLAB (Version R2020b, MathWorks Inc., Massachusetts, USA) using the FBP algorithm with a Hann filter (Ahlstrom & Tompkins, 1985 ▸). This common reconstruction algorithm was chosen because the goal of the present work was to illustrate the power of the phase-retrieval method. The sample containing more porous bone was reconstructed with a projection-specific centre-of-rotation correction, as the custom sample holder connected to the pump restricted the rotation. Reconstructions were segmented and the resulting void spaces were rendered in *Dragonfly*.

## 3. Results

### 3.1. Phase retrieval on metal test sample

We first explored propagation-based phase-contrast imaging using a Be-filtered, or pink, beam on the low-absorption metal-foil sample. Fig. 2 ▸(*a*) shows a radiograph at a sample-to-detector distance Δ = 189 mm. The sample is seen in the middle of the image. At the bottom, where the sample is thickest, the sample is easily identifiable, and the width of the sample decreases towards the top where it bends to the side. To illustrate the effect of the phase retrieval, the phase-retrieved image is compared with the negative logarithm of the normalized image, *i.e.* with −ln[*I*(*x*, *y*)/*I*_0_(*x*, *y*)], where *I*_0_ is the white-beam image, corresponding to the apparent linear attenuation.

Phase retrieval based on the assumption of a monochromatic beam yielded the image in Fig. 2 ▸(*b*), from which a clear improvement in the signal-to-noise ratio is apparent. Here noise is defined as variation in observed counts in a region where the counts are expected to be the same.

**Figure 3:**
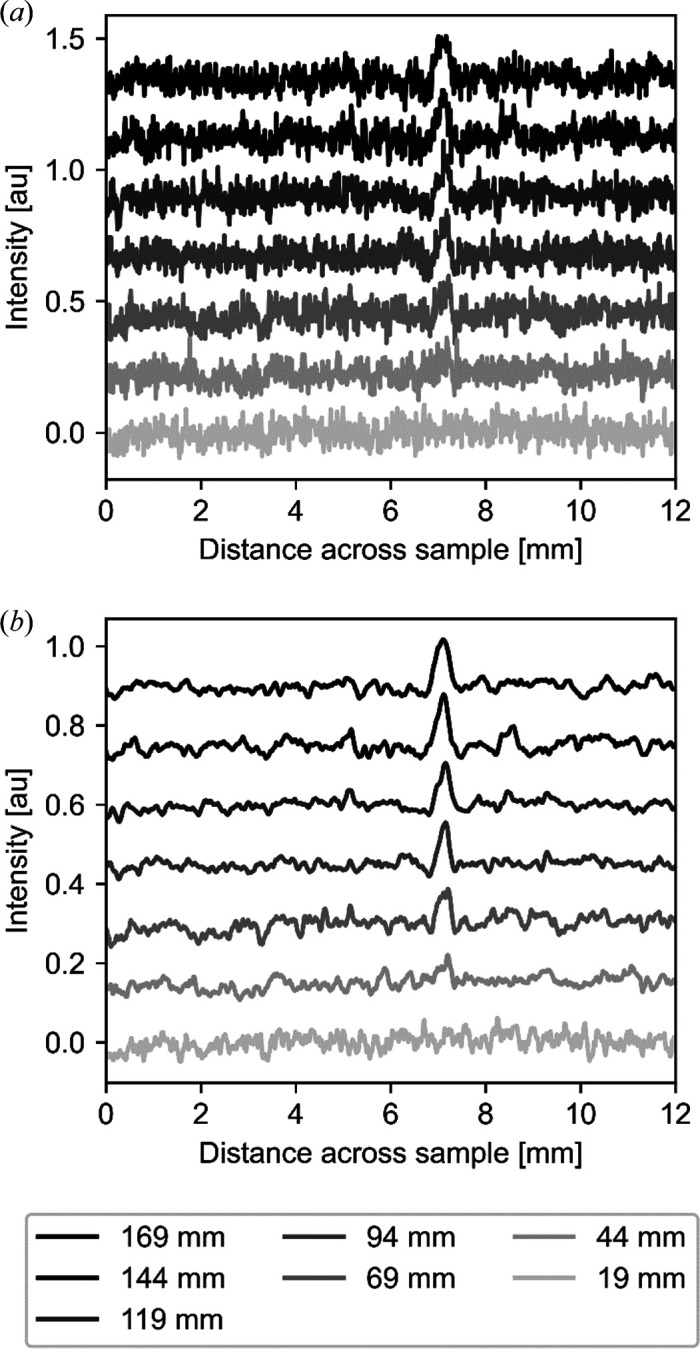
The phase-contrast signal increases for the low absorption contrast metal foil sample when increasing the sample-to-detector distance. The graphs show a vertical mean over the bottom 20 rows of pixels of the two images shown in Fig. 2 where the sample is thickest. Data at every 25 mm increase in sample-to-detector distance are shown. The graphs are offset vertically for clarity. (*a*) Data without phase retrieval and (*b*) data after phase retrieval, corresponding to Figs. 2(*a*) and 2(*b*), respectively.

To investigate systematically the effect of increasing phase contrast, we varied the sample-to-detector distance from near the detector (19 mm) in steps of 5 mm to 189 mm. Fig. 3 ▸ shows the vertical average of 20 rows of pixels across the bottom of the sample for every fifth distance measured, corresponding to every 25 mm the sample moved away from the detector.

No discernible signal was observed from this sample until a sample-to-detector distance of Δ = 69 mm [Fig. 3 ▸(*a*)]. Thus, the sample was effectively ‘invisible’ at short sample-to-detector distances due to the low absorption cross section; only upon increasing the distance, and thus increasing the phase contrast, did the sample become visible. The signal increases linearly with increasing sample-to-detector distance. This illustrates how propagation-based phase contrast builds up and reveals information almost impossible to detect using absorption contrast. Phase retrieval not only retrieves signal but also, and importantly, reduces high-frequency noise and thus increases the signal-to-noise ratio [Fig. 3 ▸(*b*)]. This also clearly demonstrates that a polychromatic neutron beam yields a phase signal. Phase retrieval for this case was performed using the Paganin single-distance phase retrieval as defined in equation (2), assuming a monochromatic beam and using the average wavelength of the beam spectrum.

**Figure 4:**
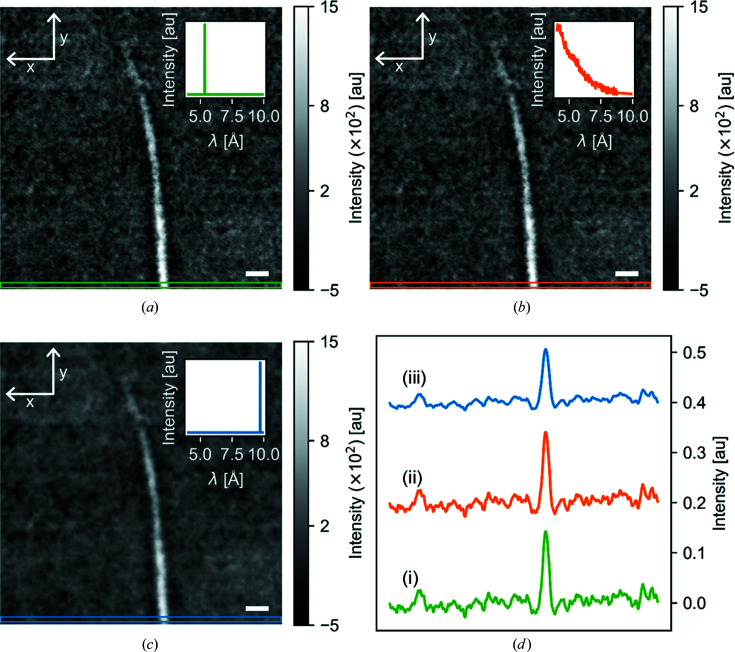
(*a*), (*b*), (*c*) Radiographs at a sample-to-detector distance of Δ = 189 mm after energy-dependent phase retrieval. The insets show the spectra that were inserted into the filter. Scale bars represent 1 mm. (*d*) Line plots showing a vertical mean over the bottom 20 rows of pixels of the images, as shown by the coloured boxes in panels (*a*)–(*c*). The data are offset along the *y* axis for clarity.

In order to compare phase retrieval using the Paganin method [equation (2)] and the generalized polychromatic version of the Paganin method [equation (5)], both were applied to the same radiography image [Figs. 4 ▸(*a*) and 4 ▸(*b*), respectively]. The insets represent the beam spectrum used in the Paganin phase retrieval. For Fig. 4 ▸(*a*), we assumed a monochromatic beam described by a δ function with a peak at the average wavelength λ = 5.2 Å, while for Fig. 4 ▸(*b*) we assumed a polychromatic beam with the spectrum shown in the inset. The two results look nearly identical, as seen from the noise patterns in the images and from comparing line cuts from the radiographs in plot Fig. 4 ▸(*d*).

This means that functionally there is no difference between using the methods defined in equation (2) or equation (5) for the phase retrieval, as long as the wavelength used in equation (2) is the average wavelength of the spectrum. Using a wavelength much higher than the average leads to overdamping and blurring of the signal, as seen in Fig. 4 ▸(*c*) and when comparing the line cuts in Fig. 4 ▸(*d*).

### 3.2. Bone results

The bone samples were investigated by X-ray microtomography prior to neutron investigations. The samples were then investigated using propagation-based phase-contrast neutron tomography, one sample with and the other without D_2_O injected into the sample. For the metal foil sample, a minimum propagation distance of 69 mm was observed, while the bone samples were placed much closer to the detector. With an exposure time of 80 s per projection, the bone samples are expected to have improved signal-to-noise ratios compared with the metal foil sample measured with 15 s exposure time. With an improved signal-to-noise ratio, the phase contrast signal is expected to be discernible at shorter distances.

**Figure 5:**
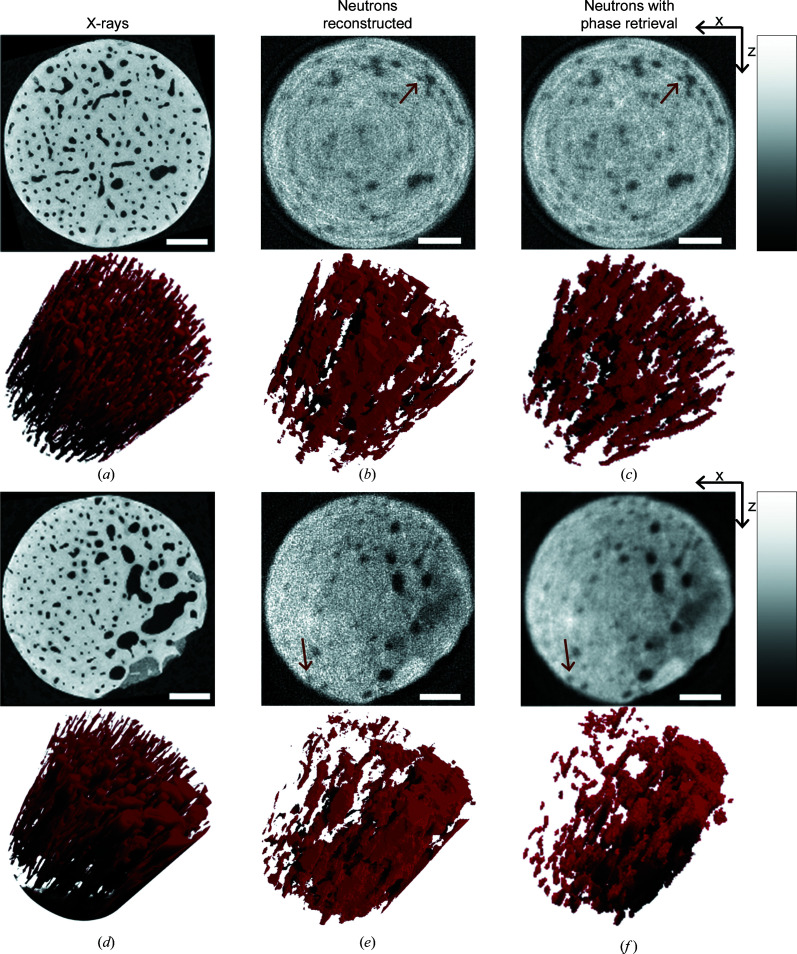
Bone samples. Typical cross sections and 3D visualizations of the void spaces for (*a*), (*d*) the laboratory-based microtomography X-ray data, (*b*), (*e*) the reconstructed neutron data and (*c*), (*f*) the phase-retrieved neutron data. The top panels show the bone sample composed of cortical bone only, while the bottom panels show the bone sample composed of more porous cortical bone. Scale bars represent 1 mm. Brown arrows point to finer features where phase retrieval enables a clearer identification from the noise. Corresponding 3D renderings of the segmented blood vessels are shown in red; images have been rotated to be displayed in the same orientation. The contrast in the images is enhanced by limiting the range of the red intensity scale from 2 to 98% of the maximum values in the data.

Fig. 5 ▸ compares virtual sections through the image stacks and rendering of void spaces from the different experiments. For the neutron tomography, it is clear that the phase-retrieval procedure improves the signal-to-noise ratios considerably, strongly improving the possibility of interpreting the data, for example via segmentations, similar to what was observed for the metal foil sample. We used the X-ray microtomography data as the ground truth for evaluating the ability to detect the vasculature in the samples. The rendered blood vessels are detectable for the phase-retrieved neutron data, but less identifiable for the raw neutron data (Fig. 5 ▸). Yet, when a comparison is made with the X-ray renderings, the difference in voxel size and signal-to-noise ratio between the two imaging modalities becomes apparent, since significantly more vascular canals were resolved with X-rays. Nevertheless, the data clearly demonstrate that, even with a white beam and a sample with significant absorption, phase retrieval via propagation-based phase contrast improves the signal-to-noise ratio significantly and provides an improved ability to identify important structural features in the material. We emphasize that the tomographic reconstructions of the neutron data were performed by simple filtered back projection, without any attempts at noise reduction or other efforts to improve the reconstruction quality. This was chosen to allow simple evaluation of the impact of phase retrieval on the signal-to-noise ratio of the reconstructed data. The reconstructed neutron data thus do not reflect the best-quality optimized reconstructions, but rather serve to illustrate the improvements attainable by phase retrieval.

**Figure 6:**
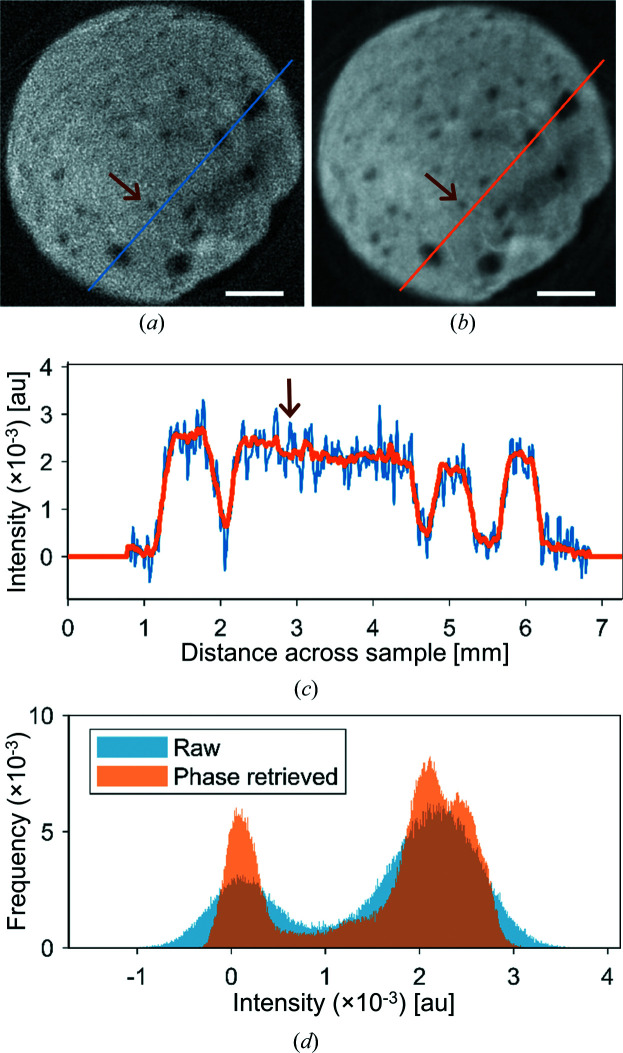
Lines through reconstructed phase-contrast neutron data. (*a*) A cross section through the reconstructed raw neutron data. (*b*) A cross section through the reconstructed phase-retrieved neutron data. Scale bars represent 1 mm. (*c*) Intensity plots of the blue line in (*a*) and the orange line in (*b*). Brown arrows point to finer features where phase retrieval enables a clearer identification from the noise. (*d*) Histograms of raw (blue) and phase-retrieved (orange) neutron data. The intensity bin width is 1 × 10^−5^.

To evaluate the improvement in signal-to-noise ratio upon phase retrieval, we chose a line going through both bone and void space and used it to compare raw and phase-retrieved data (Fig. 6 ▸). From this, it is evident that the Paganin phase-retrieval method improves the signal-to-noise ratio significantly. The Paganin phase retrieval allows for identifying finer features, which is clear from both the line cuts in Fig. 6 ▸(*c*) and the 3D segmented renderings in Fig. 5 ▸. Arrows are drawn on both these figures, pointing to finer features that are clearly discernible from the noise after phase retrieval due to the improved signal-to-noise ratio. In addition, Fig. 6 ▸(*d*) shows intensity histograms from which it is apparent that the noise level in the raw data does not allow for the separation of more than air and bone. Nonetheless, additional peaks of *e.g.* D_2_O are visible in the phase-retrieved data histogram.

**Figure 7:**
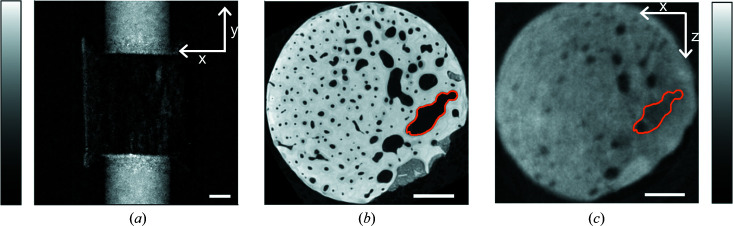
D_2_O uptake in the sample containing more porous bone. (*a*) Two raw neutron detector frames subtracted so that the D_2_O lights up on all sides of the sample. White spots mid-sample are assigned to D_2_O in bigger void spaces, corresponding to the area marked in orange in panels (*b*) and (*c*). (*b*) A typical cross section through the laboratory-based microtomography X-ray data, with a bigger void marked in orange. (*c*) A typical cross section through the phase-retrieved neutron data, with the bigger D_2_O-filled void marked in orange. All scale bars equal 1 mm.

The possibility of harnessing propagation-based phase-contrast imaging for studying liquid transport was investigated by radiography of the bone sample containing more porous bone. With an exposure time of 80 s, it only took one frame for the D_2_O to be seen on the other side of the sample. Due to imperfect sealing at the sample cell/bone interface, some D_2_O circumvented the sample, as seen by the high signals along the edges of the sample [Fig. 7 ▸(*a*)]. The sample was purposely oriented with the bigger void spaces to one side, and these void spaces light up as white spots when subtracting two detector frames [Fig. 7 ▸(*a*)], indicating the presence of D_2_O in the bigger bone pore spaces at least. A neutron propagation-based phase-contrast tomogram was collected of the sample containing D_2_O. Thus, some of the canals contained D_2_O. D_2_O and bone have quite similar intensity, making them difficult to distinguish using absorption neutron imaging (Le Cann *et al.*, 2017 ▸; Törnquist, 2021 ▸). Indeed, we could not directly segment the water-filled voids from the phase-retrieved data. However, correlative imaging combining X-ray and neutron tomograms easily allowed the identification of fluid-filled pore spaces. One of the bigger canals/voids is traced with orange marking in a slice through the X-ray image [Fig. 7 ▸(*b*)]. When this region is superimposed on the neutron slice taken after D_2_O uptake, the canal is found to be filled with D_2_O, giving rise to a density closer to bone than to air [Fig. 7 ▸(*c*)]. When comparing other canals in these two slices, it is evident that the traced canal is not the only one filled with D_2_O. This illustrates that correlative imaging combining X-ray and neutron contrast can be most helpful in analysing complex materials like bone.

**Figure 8:**
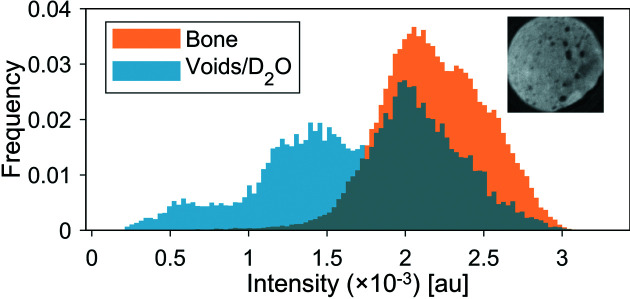
Histograms of the grey levels of bone (in orange) and of void spaces identified by X-ray microtomography (in blue), some of which are filled with D_2_O in the neutron experiment. The intensity bin width is 3 × 10^−5^.The inset shows the slice used in the histogram.

To visualize the D_2_O in the bone pores, segmentation of a slice of the X-ray data has been superimposed on the neutron data of the sample containing D_2_O, while matching the orientation and rotation of the slices as well as possible. Histograms of the grey-level data in the segmented voids and bone are plotted in Fig. 8 ▸. Three peaks are present in the histogram, one at the same intensity as the bone peak and two at slightly lower intensities, corresponding to D_2_O and air. As the resolution of the neutron data is lower than that of the X-ray data, many of the voids segmented from the X-ray data are not present in the neutron data, giving rise to the relatively large bone peak in the void histogram. The few voids that are present and filled with air in both data sets give rise to the peak at the lowest intensity in the histogram. The peak at the intermediate intensity therefore corresponds to the D_2_O present in the larger void spaces in the sample, indicating the successful penetration of D_2_O into the sample.

## 4. Discussion

Propagation-based phase-contrast neutron imaging was recently demonstrated to work on a biological sample using near-monochromatic neutrons (Paganin *et al.*, 2023 ▸). Here we wished to test whether the methodology can also be employed when using polychromatic neutron radiation. We have proved the viability of the Paganin phase-retrieval method with a polychromatic beam on a metal foil and a bone sample. The results from the metal foil sample showed that the generalized phase-retrieval method for a polychromatic beam using a spectrally averaged neutron cross section is functionally no different from using the method for a monochromatic beam, provided the wavelength inserted into the method is the weighted mean of the neutron spectrum. This is relevant when imaging materials for which the energy-dependent neutron cross section is not known *a priori*.

For both the metal foil and bone samples, an amplification of the signal-to-noise ratio was observed, indicating that phase retrieval can be used to reduce the acquisition time and improve the image contrast and/or spatial resolution of low-absorption materials (Paganin *et al.*, 2023 ▸). A potentially larger increase in spatial resolution can be achieved with interferometric methods, which measure absorption contrast, phase contrast and dark-field contrast (Pushin *et al.*, 2017 ▸; Strobl *et al.*, 2019 ▸), but it comes at the expense of an increase in measurement time. This makes neutron interferometry less suitable for *in situ* experiments where time resolution is of the essence.

Neutron and X-ray radiation have very different relative scattering cross sections for the elements in the periodic table. Whereas the X-ray scattering cross section is almost proportional to the electron density, the neutron cross sections fluctuate and are independent of atomic number. Hence, X-ray and neutron radiation generally lead to very different imaging contrast. Combining the two contrast methods for the same sample therefore allows a more comprehensive study than can be achieved using only one modality, as previously discussed by Törnquist *et al.* (2021 ▸) and Guillaume *et al.* (2021 ▸) and now demonstrated in the present study.

X-ray radiation provides good contrast between bone, soft tissue and air. For studies of highly X-ray absorbing metal implants in bone, for example, neutrons can be very useful, since the different contrast avoids metal artefacts stemming from the highly absorbing implant which strongly impedes standard X-ray imaging (Le Cann *et al.*, 2017 ▸; Isaksson *et al.*, 2017 ▸).

Liquid transport through a piece of bone is another example of the usefulness of neutrons. For this type of experiment, we ideally wish to study the transport of a liquid through a biological material. With X-rays, it would be necessary to use contrast agents not part of the normal biological environment. With neutrons, D_2_O can provide the contrast needed, while being close to the biological scenario. Secondly, mixtures of D_2_O and H_2_O can be used to adjust the contrast of the fluid. In the present study, we found that combining data from X-ray and neutron sources provides more information about the samples than is available from either modality on its own. With the phase-retrieval algorithm, neutron phase-contrast imaging is a good candidate for investigating bone and other low-absorption materials where the goal is to distinguish different materials with similar X-ray contrast. Even though we observed that parts of the liquid circumvented the sample due to insufficient sealing between the custom sample holder and the bone sample, the *in situ* experiment still demonstrated the viability of the phase-retrieval approach.

## 5. Conclusions

Propagation-based phase-contrast neutron imaging has been successfully demonstrated on both a metal sample with very low absorption contrast, *i.e.* an almost phase pure object, and a biological sample, here bone. The results indicate that this method, in combination with the Paganin single-distance phase-retrieval method, helps decrease noise and increase contrast, which makes it a good method for low-attenuation samples and likely to work well with *in situ* experiments.

Combining neutron and X-ray radiation for investigating bone and other biological or hierarchical materials provides an increase in complementary information compared with a single modality.
